# Tips and tricks for a safe and effective image-guided percutaneous renal tumour ablation

**DOI:** 10.1007/s13244-017-0555-4

**Published:** 2017-05-12

**Authors:** Giovanni Mauri, L. Nicosia, G. M. Varano, G. Bonomo, P. Della Vigna, L. Monfardini, F. Orsi

**Affiliations:** 1 Divisione di Radiologia Interventistica, Istituto Europeo di Oncologia, Via Ripamonti 435, 20141 Milan, Italy; 2 Servizio di Radiologia, IRCCS Policlinico San Donato, Piazza Edmondo Malan, San Donato Milanese, Italy; 3 Scuola di specializzazione in Radiodiagnostica, Università degli studi di Milano, Via Festa del Perdono 7, Milan, Italy; 4 Dipartimento di radiologia e diagnostica per immagini, Poliambulazna di Brescia, Via Leonida Bissolati 57, Brescia, Italy

**Keywords:** Renal tumours, Renal ablation, Protective manoeuvres, Image-guided tumour ablation, Image guidance

## Abstract

**Abstract:**

Image-guide thermal ablations are nowadays increasingly used to provide a minimally invasive treatment to patients with renal tumours, with reported good clinical results and low complications rate. Different ablative techniques can be applied, each with some advantages and disadvantages according to the clinical situation. Moreover, percutaneous ablation of renal tumours might be complex in cases where there is limited access for image guidance or a close proximity to critical structures, which can be unintentionally injured during treatment. In the present paper we offer an overview of the most commonly used ablative techniques and of the most important manoeuvres that can be applied to enhance the safety and effectiveness of percutaneous image-guided renal ablation. Emphasis is given to the different technical aspects of cryoablation, radiofrequency ablation, and microwave ablation, on the ideal operating room setting, optimal image guidance, application of fusion imaging and virtual navigation, and contrast enhanced ultrasound in the guidance and monitoring of the procedure. Moreover, a series of protective manoeuvre that can be used to avoid damage to surrounding sensitive structures is presented. A selection of cases of image-guided thermal ablation of renal tumours in which the discussed technique were used is presented and illustrated.

***Teaching points*:**

• *Cryoablation, radiofrequency and microwave ablation have different advantages and disadvantages.*

• *US, CT, fusion imaging, and CEUS increase an effective image-guidance.*

• *Different patient positioning and external compression may increase procedure feasibility.*

• *Hydrodissection and gas insufflation are useful to displace surrounding critical structures.*

• *Cold pyeloperfusion can reduce the thermal damage to the collecting system.*

## Introduction

Kidney cancer are among the most prevalent tumours in western countries, with an incidence of 2,9–15 per 100,000 in Europe and 50,000 new cases in the United States each year [1–3]. Conventional treatment has been historically represented by radical nephrectomy, while, more recently, surgical nephron sparing techniques have been developed in order to reduce the invasiveness of the treatment [4–6]. For the same reason, image-guided thermal ablations have been successfully applied in several different organs, including liver, lung, bone, and even neck tumours [5, 7–14]. The necessity to offer a treatment to patients not suitable for surgery, and to spare the highest amount of normal renal parenchyma has pushed for the application of image-guided ablative techniques to renal cancer [15, 16] with reported good clinical results [17–19]. However, some factors could limit the feasibility and/or safety of these techniques. In particular, the proximity to the tumour of sensitive structures that might be damaged by thermal injury can limit the application of image-guided ablations [20].

The availability of the most advanced imaging techniques, including a dedicated room with computed tomography (CT) and a last generation ultrasound (US) machine, possibly equipped with fusion imaging, may enhance the correct targeting of the tumour and maximize the technical result [21–25]. Furthermore, different ablative techniques might be used, each one with its own specific characteristics that should be taken into account in order to minimize the risk of complications. Moreover, the application of some protective manoeuvres, such as hydrodissection, gas insufflation, external compression, device bending and retrograde pyeloperfusion are useful to overcome these limitations. A good knowledge of differences among ablative techniques, optimal room setting and image guidance, and of possible protective manoeuvre is of paramount importance for a safe and effective application of image guided ablation to renal tumours.

Thus, in the present paper we offer an overview of the most frequently used ablative techniques, the best image guidance setting and of the most important protective manoeuvres that can be applied to image-guided thermal ablation of renal tumours, also presenting some didascalic cases.

### Ablative techniques

The most widely used ablative techniques used to treat renal tumours are cryoablation, radiofrequency ablation, and microwave ablation [9, 26–41] .

With cryoablation, repeated freeze-thaw cycles determine extracellular and intracellular ice formation with subsequent injury to cell membranes [26–28]. Cryoablation provide good visualization of the ice ball with both US and CT, which facilitates precise control of the procedure. Furthermore, as ice forming during the ablation firmly sticks to the surrounding tissues, it is possible to perform a gentle torqueing in order to displace the tumour from sensitive structures. Finally, as with cryoablation, the collagenous architecture is preserved, this technique appears to be ideal in tumours close to the calyx, as it might reduce the risk of fistula [29]. On the contrary, as cryoablation does not provide the “coagulative effect”, this technique is burdened by an higher bleeding risk than other techniques [30–32].

With radiofrequency an alternating current determines ionic friction that leads to slow heat generation, with subsequent protein denaturation, blood coagulation, and coagulative necrosis [33, 34]. RFA determines also blood coagulation, thus reducing the bleeding risk in comparison with cryoablation [35]. Moreover, with a dedicated umbrella-shaped device it is possible to stick the electrode to the tissue. One of the most important limitations of RFA is represented by the loss of heat occurring close to vessels due to the blood flow (heat sink effect). However, heat propagation from the tip of the electrode might determine injury to close sensitive structures [37, 38].

With the microwave technique the ablative antenna generates an electromagnetic radiation that produces oscillation of water molecules with formation of frictional heating, achieving very high temperature in very short time, with larger and faster ablations [39]. This technique seems to be less affected by the heat sink effect that limits radiofrequency. However, as temperatures higher than with radiofrequency can be rapidly obtained, particular caution should be used for tumours abutting the renal sinus, as urine boiling can be fast obtained with subsequent damage on the collecting system [40, 41].

## Operating room setting, optimal image guidance, and ideal patient management

**Fig. 1:**
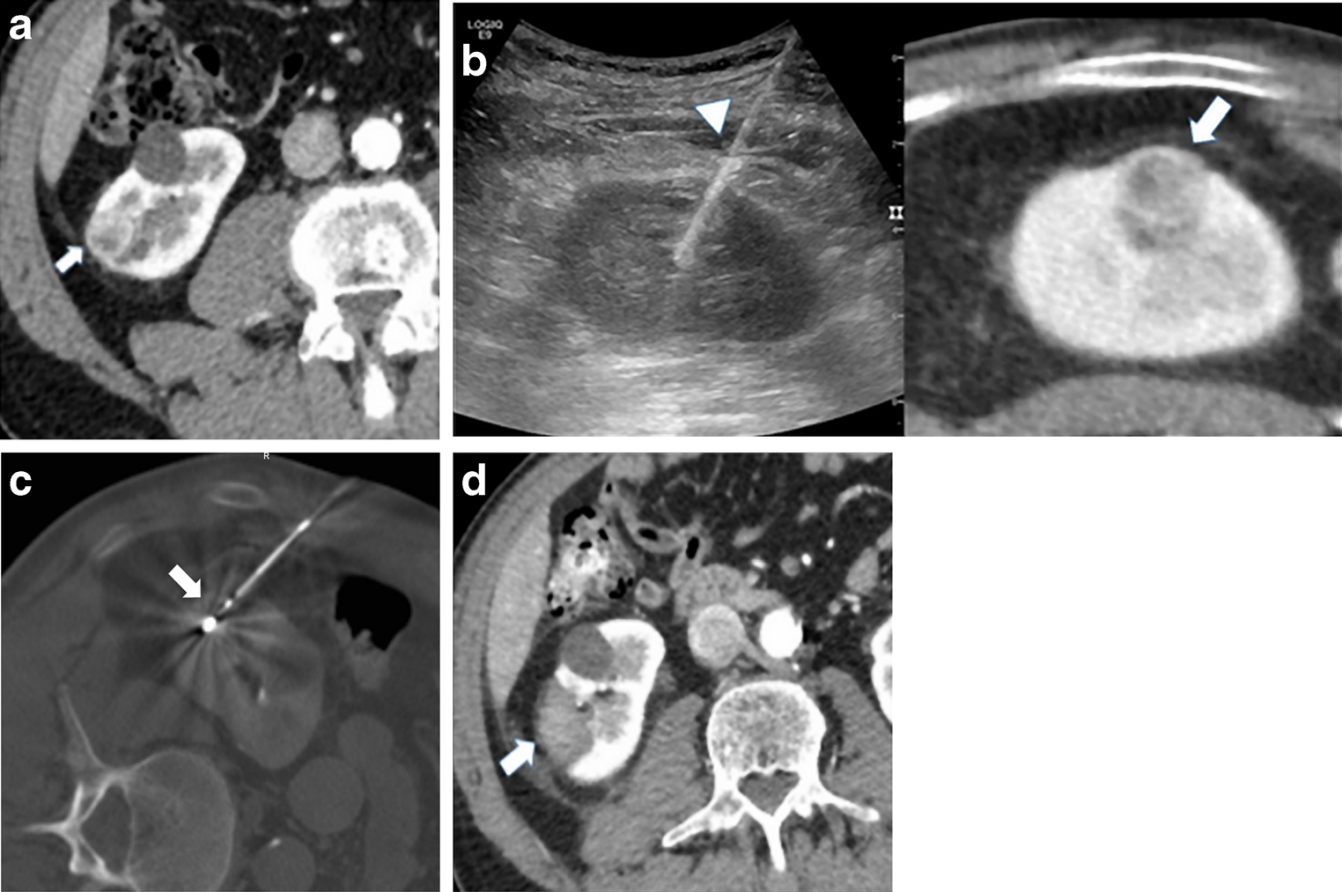
Treatment of a right renal tumour with the application of US/CT fusion imaging. **a** Contrast enhanced CT demonstrating a right renal tumour (*arrow*). **b** needle insertion was performed with fusion of real-time US with the preacquired CT that allowed the precise positioning of the microwave antenna (*arrowhead*) at the level of the tumour (*arrow*). **c** CT acquisition immediately after antenna insertion confirmed the correct positioning of the device (*arrow*) in the desired position. **d** contrast enhanced CT 24 h after treatment demonstrated the correct ablation of the tumour (*arrow*)

**Fig. 2:**
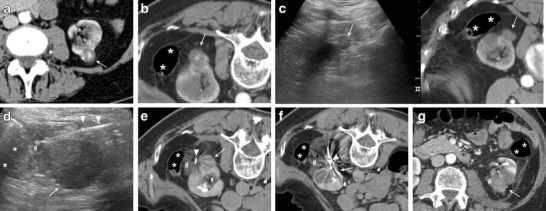
Microwave ablation of a left posterior renal cell carcinoma close to the colon with hydrodissection. **a** Contrast enhanced CT demonstrating a left posterior renal cell carcinoma (*white arrow*) to be treated with percutaneous thermal ablation. **b** Contrast enhanced CT performed the day of the procedure. To achieve a safe path to the tumour the patient is placed in prone position with external compression. In this position, the colon (*asterisks*) appears to be close to the tumour to be treated (*white arrow*). **c** fusion of contrast enhanced CT and real-time US allowed for the identification of the tumour to be treated with US (*white arrow = tumour; asterisks = colon*). **d** under US guidance a small spinal needle (*arrowheads*) is placed in between the tumour (*white arrow*) and colon (*asterisks*) and fluid (*hash*) is injected. **e** A CT scan performed after fluid (*hash*) injection confirmed the correct displacement of the colon (*asterisks*) from the target tumour (*white arrow*) (*arrowhead = spinal needle*). **f** CT scan demonstrating the insertion of the microwave antenna (*arrowhead*) into the renal tumour (*white arrow*) (*asterisks = colon; hash = injected fluid*). **g** Contrast-enhanced CT performed the day after treatment demonstrating the complete ablation of the renal tumour (*white arrow*)without complications (*asterisks = colon*)

Several imaging modalities can be used for guiding percutaneous tumour ablation, the two most widely used being US and CT. US is a radiation-free imaging modality, providing high-resolution real-time imaging, allowing for non-axial imaging, and for continuous monitoring of the different phases of the treatment. Immediate post-treatment evaluation with contrast-enhanced US (CEUS) can also be performed [23]. However, US is burdened by limitations in the case of obese patients, presence of air (e.g. intestinal loops), and often is lacking in contrast resolution particularly in cases of iso-echoic renal tumours. Conversely, CT provides a larger field of view and a better visualization of renal tumours and structures containing air. However, CT requires use of ionizing radiation, provides mainly an axial view, and contrast media usage has to be cautiously applied. Thus, ideally, both modalities should be available in the operating theater. Systems that allow for the acquisition of CT images and real-time fusion with US seems to be extremely beneficial for image guided tumour ablation, allowing for merging the advantages of both the two imaging modalities and overcoming their limitations [22, 23]. These types of systems have been already applied to the treatment of liver tumours, even for liver lesions completely invisible at US [22]. In renal ablation, such techniques provide evident advantages in the identification of the lesion, which is often not conspicuous at US. At the same time, the possibility of controlling in real time the needle insertion with US, with constant reference to a co-registered CT, enhances the safety of the procedure. However, since the kidney is highly mobile, displacement of the organ during the procedure might occur, reducing the reliability of the fused CT images. Thus, fusion imaging cannot be used as the sole method of guidance, and US should always be considered the reference for device insertion. Two cases are shown in Figs. 1 and 2.

Particularly helpful is to perform renal ablations under general anesthesia. General anesthesia allows for avoiding patients’ movements during the procedure and even achieves a controlled breath-hold that enhances the targeting of the tumour and decrease the risk of unintended puncture of undesired structures.

After the procedure it is always recommended to perform a contrast enhanced CT to control for immediate result and eventual presence of complications.

## Patient positioning and external compression

Patients’ position change might be sometimes enough to obtain a sufficient distance between the tumour and the surrounding structure to perform a safe ablation. For this purpose, careful planning, and application of dedicated devices such as vacuum mattress are of paramount importance. A simulation can be made performing CT in different positions before the procedure. This can be done the same day of the procedure or some days in advance, changing the position of the patient up until the identification of the best and easiest trajectory for the ablative device.

## Hydrodissection

**Fig. 3:**
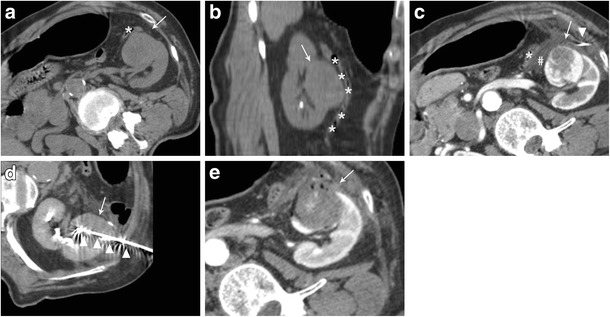
Treatment of an anterior left renal tumour in close proximity with a bowel loop**. a** CT performed the day of treatment showing the close proximity of the tumour to be treated (white arrow) with a bowel loop (asterisks). Note the external compression to obtain the desired position of the patient for an easy access to the target tumour. **b** multiplanar reconstruction of the CT images showing the close proximity of the tumour (white arrow) with a bowel loop (asterisks). c A CT scan performed after fluid (*hash*) injection confirmed the correct displacement of the colon (asterisks) from the target tumour (*white arrow*) (*arrowhead = spinal needle*). d CT scan demonstrating the insertion of the microwave antenna (*arrowhead*) into the renal tumour (*white arrow*). e Contrast-enhanced CT performed immediately after treatment demonstrating the complete ablation of the renal tumour (*white arrow*) without complications

Sensitive structures close to the target tumour may be displaced by injecting different substances in between. In particular, when a radiofrequency ablation with hydrodissection is planned, saline solution should be avoided because of its high electrical conductivity, and other fluid solution, such as glucose solution, have to be used. To perform hydrodissection a small needle is advanced between the tumour itself and the structure to be displaced. Generally, the instillation of 135–150 cm^3^ might be enough to displace adjacent bowel loops by about 2.1–2.5 cm [42]. Imaging is generally repeated after injection of the initial bolus of fluid to confirm deposition into the desired location. The needle can be left in place for subsequent instillation during thermal ablation [43]. Two cases are shown in Figs. 2 and 3.

## Gas insufflation

Some authors applied injection of normal air [44], while others reported the use of carbon dioxide (CO2) for organ displacement [45, 46]. CO2 seems to be particularly useful, as it is quickly reabsorbed by the body and eliminated by respiration. CO2 has a low thermal conductivity, and large volumes can be safely injected.

**Fig. 4:**
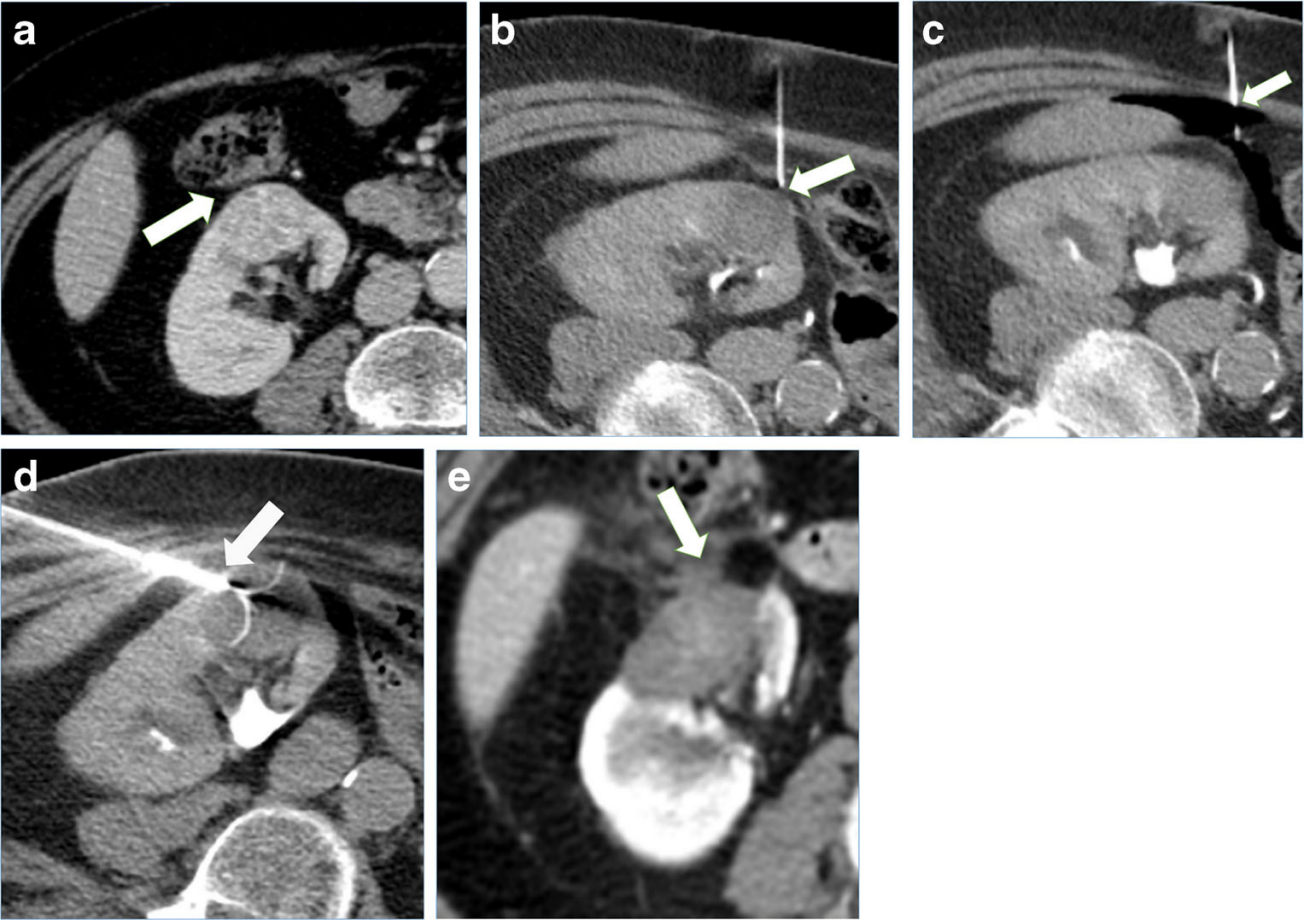
Treatment of a right renal tumour with image guided thermal ablation with the assistance of CO2 injection. **a** contrast enhanced CT demonstrating an anterior right renal tumour close to the colon (*arrow*). **b** patient was positioned in lateral decubitus and a small needle was inserted in between the tumour and the colon (*arrow*). **c** CO2 was injected through the small needle (*arrow*) in order to achieve colon displacement. **d** an umbrella-shaped radiofrequency electrode (*arrow*) was inserted into the tumour from the opposite site of the colon, and slight traction made in order to displace the organ. **e** a contrast enhanced CT performed 24 h after the ablation demonstrated good ablation of the tumour (*arrow*) without complications

Because of its nature, CO2 cannot be seen with US, thus, after careful needle placement in the desired position and after CO2 injection, the ablation procedure is often followed by CT imaging [45, 47]. Since it is lighter than fluid, CO2 tends to diffuse anteriorly, and thus CO2 injection appears to be particularly useful to achieve displacement of structures that are located anteriorly to the tumour to be treated. A case is shown in Fig. 4.

## Electrode torqueing

This technique can be used mainly with umbrella-shaped radiofrequency devices and with cryoablation. The tip of the ablation device is anchored into the tumour, and small movements of the organ can be achieved by gentle traction. It can be used in adjunct to other methods, such as hydrodissection or gas insufflation, to increase the distance between the tumour and surrounding structures. Great caution has to be used, as forceful torqueing can potentially cause vascular or parenchymal injuries of the organ [42, 48]. An example is shown in Fig. 4.

## Balloon interposition

This is usually a second-line technique: after abdominal wall puncture with an 18 or 19G coaxial needle, a 0.035-in. wire is placed trough the needle. A sheath is then advanced over the wire, and a balloon is advanced over the wire and trough the sheath. Once the position is adjusted under image guidance, the balloon is expanded completely [49, 50].

## Cooled pyeloperfusion

**Fig. 5:**
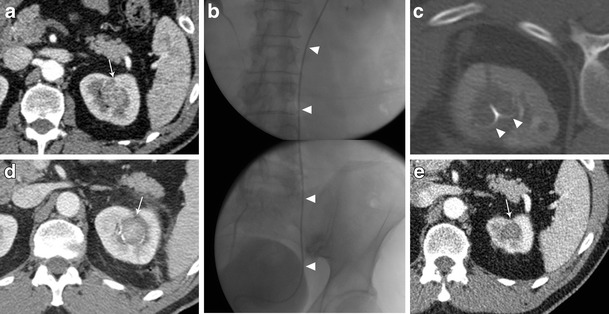
Treatment of a left kidney central tumour with renal sinus extension. **a** contrast enhanced CT demonstrating a centrally located tumour (*white arrow*) with extension in close proximity of the renal sinus. **b** retrograde pyeloperfusion was performed through a single j stent (*arrowheads*) placed endoscopically the day of the ablation. **c** CT scan after insertion on a radiofrequency electrode (*arrowheads*) into the tumour. **d** contrast enhanced CT performed immediately after the ablation demonstrated the complete ablation of the tumour (*white arrowhead*) without complications. **e** Contrast enhanced CT 2 years after the ablation demonstrating complete ablation with tumour shrinkage and no complications

In order to protect the collecting system from thermal damage, perfusion of the system with cold fluid solution can be performed. For this purpose, a retrograde ureteral 6 Fr single J catheter is placed before the ablation, and refrigerated solution is infused during the treatment. A small catheter is used in order to facilitate the water circulation into the renal pelvis. The ureteral single J is connected to a three-way stopcock, one way connected to the cold fluid bag and another to an empty collecting bag. Pyeloperfusion is performed intermittently by opening and closing the three-way stopcock [51]. A case is shown in Fig. 5. The catheter is left in place, secured to a bladder catheter, up to the day after the procedure. In cases where a fistula is demonstrated at subsequent 24-h control, the catheter is left in place for a number of days and removed after subsequent imaging control and demonstration of resolution.

## Conclusions

During the last few years, percutaneous thermal ablation has become a widespread alternative to nephrectomy and nephron sparing surgery thanks to the good clinical results, low invasiveness, low morbidity and rapid patient recovery.

Although percutaneous thermal ablation is considered safe, it can be complicated with unintended thermal injury to the surrounding structures, such as bowel loops, nerves, or the urinary collecting system.

Interventional radiologists who perform renal thermal ablation should have a fully equipped operating theater, possibly including CT, US, and a system for fusion imaging, and they should be familiar with all the ablative techniques and protective manoeuvres that can be applied to maximize the result and reduce complications.
